# Supplementary material for: Structural and functional analysis of the roles of the HCV 5′ NCR miR122-dependent long-range association and SLVI in genome translation and replication
Source: PeerJ. 2018 Nov 6;6:e5870. doi: 10.7717/peerj.5870 (PMC6225842; doi:10.7717/peerj.5870)

Fig. S1. Folding predictions for JFH1-CEtrans plus/minus miR122. (A) Predicted structure of JFH1-CEtrans in the absence of miR122 binding generated using mfold prediction software. Structure shows the formation of the LRA with the 5' basal of stem of SLVI base paired with complementary nucleotides of the miR122 binding site in the 5'UTR. Predictions were computed from sequence in which the nucleotides comprising SLII-V were represented by a series of n's to simplify the folding. dG = -52.80 (B) Predicted structure of JFH1-CEtrans bound to one molecule of miR122 generated using bifold prediction software. Structure shows miR122 bound to seed site 1, blocking LRA formation, and the resulting SLVI structure. Predictions were computed from sequence in which the nucleotides comprising SLII-V were represented by a series of x's to simplify the folding. dG = -66.7. Blue and yellow highlighted sequences show the S1 and 5' SLVI sequences respectively, which overlap in the miR122:S1 and S1:SLVI binding motifs.

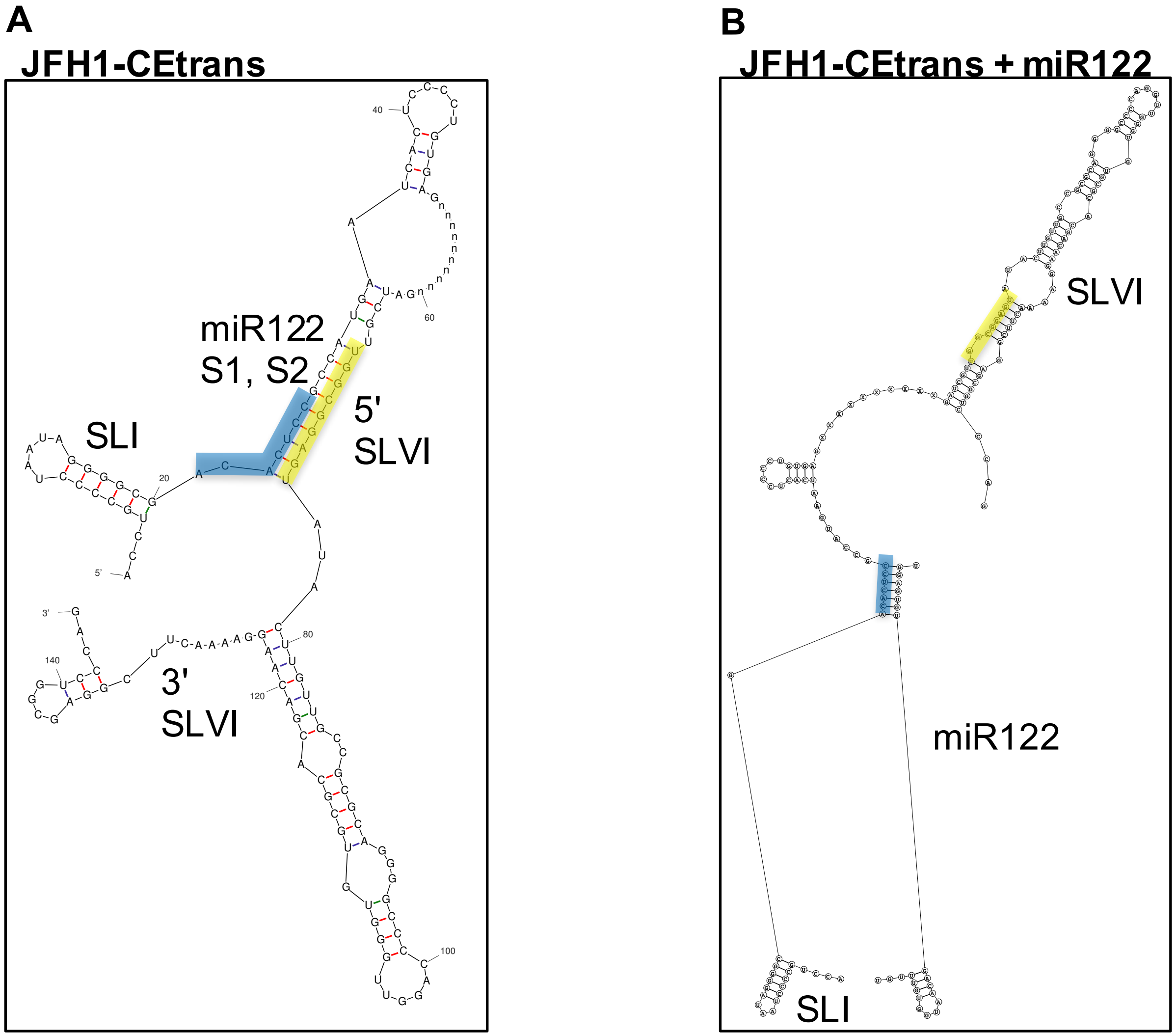

Supplement: Supplemental Information 1 — (A) Predicted structure of JFH1-CEtrans in the absence of miR122 binding generated using mfold prediction software. Structure shows the formation of the LRA with the 5’ basal of stem of SLVI base paired with complementary nucleotides of the miR122 binding site in the 5’UTR. Predictions were computed from sequence in which the nucleotides comprising SLII-V were represented by a series of n’s to simplify the folding. dG = −52.80 (B) Predicted structure of JFH1-CEtrans bound to one molecule of miR122 generated using bifold prediction software. Structure shows miR122 bound to seed site 1, blocking LRA formation, and the resulting SLVI structure. Predictions were computed from sequence in which the nucleotides comprising SLII-V were represented by a series of x’s to simplify the folding. dG = −66.7. Blue and yellow highlighted sequences show the S1 and 5’ SLVI sequences respectively, which overlap in the miR122:S1 and S1:SLVI binding motifs. [file peerj-06-5870-s001.pdf]
