# Supplementary material for: Structural and functional analysis of the roles of the HCV 5′ NCR miR122-dependent long-range association and SLVI in genome translation and replication
Source: PeerJ. 2018 Nov 6;6:e5870. doi: 10.7717/peerj.5870 (PMC6225842; doi:10.7717/peerj.5870)

Fig. S2. SHAPE analysis of JFH1-CEtrans in the presence of 10 mM $MgCl_2$. SHAPE reactivities are shown for JFH1-CEtrans plus miR122 when folded in a reaction buffer containing 10 mM $MgCl_2$, with predicted (P) and observed (O) conformations given top right below template name. Black bars show normalised SHAPE reactivities of nucleotides 427-447 and 487-507, encompassing the 5' and 3' basal stems of SLVI respectively. Nucleotides with a reactivity of <0.4 are considered unreactive and therefore base-paired. Shaded regions highlight nucleotides of importance in determining 'open' or 'closed' conformations: specifically the 5' $G_{434}G_{435}$ motif and 3' nucleotides 494-507. The superimposed red line indicates the exposure of JFH1-CEtrans plus miR122 in 5 mM $MgCl_2$ folding buffer, and is included for comparison of reactivities to a demonstrated 'open' conformation. A maximum negative reactivity was set at -0.1. Data was derived from one replicate folding reaction.

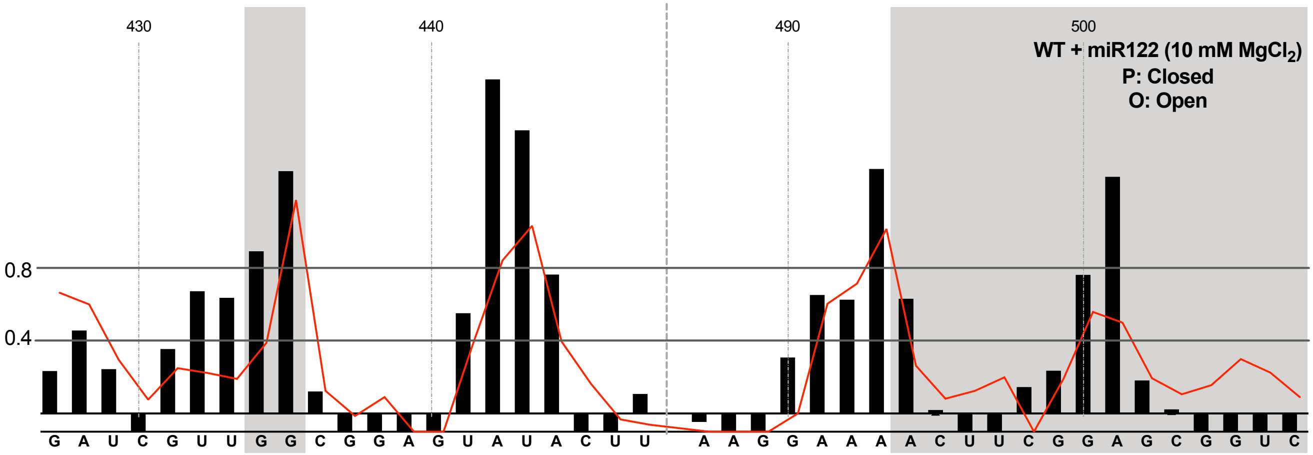

Supplement: Supplemental Information 2 — SHAPE reactivities are shown for JFH1-CEtrans plus miR122 when folded in a reaction buffer containing 10 mM MgCl2, with predicted (P) and observed (O) conformations given top right below template name. Black bars show normalised SHAPE reactivities of nucleotides 427–447 and 487–507, encompassing the 5’ and 3’ basal stems of SLVI respectively. Nucleotides with a reactivity of <0.4 are considered unreactive and therefore base-paired. Shaded regions highlight nucleotides of importance in determining ‘open’ or ‘closed’ conformations: specifically the 5’ G434G435 motif and 3’ nucleotides 494-507. The superimposed red line indicates the exposure of JFH1-CEtrans plus miR122 in 5 mM MgCl2 folding buffer, and is included for comparison of reactivities to a demonstrated ‘open’ conformation. A maximum negative reactivity was set at -0.1. Data was derived from one replicate folding reaction. [file peerj-06-5870-s002.pdf]
